# Cognitive Components of Vocal Communication: A Case Study

**DOI:** 10.3390/ani8070126

**Published:** 2018-07-23

**Authors:** Charles T. Snowdon

**Affiliations:** Department of Psychology, University of Wisconsin, 1202 West Johnson Street, Wisconsin, MI 53706, USA; snowdon@wisc.edu

**Keywords:** cognition, communication, babbling, development, dialects, food calls, pygmy marmosets, sound localization, vocalization

## Abstract

**Simple Summary:**

The pygmy marmoset is the world’s smallest true monkey with a mean brain size of 4.5 g. I review the cognitive and pre-linguistic skills that these monkeys show in their vocal communication. Pygmy marmosets have several types of calls used to maintain contact with one another, and they use calls that are cryptic when they are close to group members, and calls that more easily detected when they are farther away. They take turns in contact calling. They modify their call structure when in a new social setting to match the structure of other group members or of mates. Infants display babbling behavior similar to human infants, and adult caretakers provide social reinforcement that lead infants to develop adult like calls. Thus, pygmy marmosets modify vocal structure and learn through social interactions. This contrasts sharply with the general view that most nonhuman primates do not display vocal learning or an ability to modify call structure. Pygmy marmosets signal other group members when they find food using specialized calls but inhibit these calls with living prey. These complex cognitive skills in communication indicate that brain size may not be a good predictor of cognitive ability.

**Abstract:**

Communication among nonhuman animals is often presented as rigid and inflexible, reflecting emotional states rather than having any cognitive basis. Using the world’s smallest monkey, the pygmy marmoset (*Cebuella pygmaea*), with the smallest absolute brain size amongst simian primates as a case study, I review the role of cognition in the development and usage of vocalizations in pygmy marmosets and present new data on the instrumental use of babbling and of food associated vocalizations. Pygmy marmosets have several contact calls that differ in the psychoacoustic properties for sound localization as well as the distance at which they carry through the rainforest. Marmosets use these calls strategically based on distance from neighbors. Marmosets alter spectral and temporal aspects of call structure when exposed to new groups and when newly mated. They display population specific vocal dialects. Young pygmy marmosets engage in extensive babbling behavior rewarded by parents that helps the young develop adult vocal structures, but older monkeys also use babbling instrumentally in conflict situations. Specific food referential calls generally relate to food preferences, but food calls are suppressed in the presence of animate prey. Unmated animals systematically combine a long distance call with food calls as though advertising for mates. Taken together, these examples show that even small brained primates use their vocal signals flexibly and strategically in response to a variety of environmental and social conditions.

## 1. Introduction

Animals communicate, but what do they communicate about? Why do they communicate at all since vocal communication signals make animals more easily detected by predators? Visual signals may not be as easily detected by predators but are ineffective in dense vegetation or under poor light conditions often seen in dense tropical forests. Thus, vocal signals are important. The most common explanation is that animals communicate information that is useful to recipients. This could include information about breeding status, sex, identity, or population as well as where food is located, that predators have been found, that a good shelter or nest site has been located, or that one’s dominance or submission to another is acknowledged [1]. We do not need to attribute intentionality to a communicator. A recipient can infer information about the social or emotional state of a caller or about the presence of food or predators without any intentionality on the part of the communicator. Both the communicator and recipient, especially those living in close social groups, are presumed to benefit from an honest exchange of information that leads to increased reproductive success for all.

An alternative explanation is that animal signals have evolved to manipulate or manage the behavior of others. Under this view the physical structures of signals have a direct behavioral or physiological effect on listeners. The communicator may or may not be communicating accurate information about its own state or behavior, and the benefit of communicating is to alter the behavior of recipients [2] to the benefit of the caller. In the closely-related assessment-management model [3] animals continually assess what group mates are doing and attempt to manage the behavior of others. According to the information model animals communicate about predators, food, dominance status, and internal emotions, calls can be sorted according to their social function: alarm, arousal, avoidance, affiliation, etc. According to the manipulation or management perspective calls are sorted according to the effects they have on listeners: inducing fear, avoidance, approach, and soliciting mating.

Examples of the information approach include alarm calls given by several species that indicate a specific predator type [4,5] or at least distinguish between aerial versus ground predators [6] leading to different adaptive behavioral responses by recipients. Examples of the management approach include the sounds used by humans to control working animals [7,8] and to control nonverbal human infants [9]. Humans use similar sounds to arouse, calm, or stop action in both working animals and babies, and these likely do not reflect the motivational state of the caller, but are explicitly intended to induce behavioral changes in the listeners.

However, although distinctions can be made between these two explanations of communication, in reality, it can be difficult to make clear distinctions. An alarm call that indicates the presence of a certain type of predator also may induce fear and behavioral change in the recipient. Mobbing calls that elicit group approach to chase away a predator are short, broad band calls similar to those that induce arousal in infants and working animals, and alarm calls that lead to freezing behavior are similar to those that reduce physiological activity, yet these calls also may communicate information about the location of or urgency of response to a predator.

Vocal communication can also be described in terms of the acoustic structure of signals used and how well they can be transmitted in different environments [10], and they can also be described in terms of their social and emotional aspects [11]. An additional interest, especially with nonhuman primates, is whether the study of primate vocalizations can inform us about the evolution of language [12,13]. The vocal communication of nonhuman primates has been an apparent anomaly because early work showed that nonhuman primates do not show vocal learning, have limited flexibility in the structure and usage of their calls and do not have a vocal tract capable of producing human like sounds [14,15,16].

However, recent work has changed this view with new evidence supporting vocal learning, the ability of primate vocal tracts to produce human like speech sounds, take turns, communicate about environmental events, and adapt vocal production for strategic purposes [12,13,17]. Most of the research on the cognitive and pre-linguistic functions of vocal communication has been focused on great apes and Old World primates since these species are closer in phylogeny to humans than other species.

Not only are these species closer in phylogeny, they also have larger brains than other nonhuman primates. Many authors have related absolute brain size [18] or encephalization quotient (EQ, cortex as a function of total brain weight) to social and cognitive complexity [19]. In general there is a good correlation between body weight and EQ (re-analyses in a previous study [20] (Figures 3.2 and 3.3), derived from a previous study [21]) but some interesting anomalies exist. Humans are notable exceptions with EQ’s much larger that body size would predict. In addition, two New World primate genera (squirrel monkeys, *Saimiri* and capuchin monkeys, *Cebus*) have larger EQ than any Old World primate or ape. Dunbar [19] has argued that grooming time is related to both group size and EQ. Although grooming time and group size are positively related for Old World monkeys and apes, there is a negative relationship between grooming and group size in New World primates. Furthermore, although there is a positive relationship between group size and EQ in New World primates and apes, there is no correlation between group size and EQ in Old World primates when arboreal species are included.

What are we to make of these results? Brain size or EQ may not be adequate for models of communication complexity, nor can phylogenetic proximity to humans be considered as a predictor of brain size or EQ. Furthermore, the negative relationship between group size and grooming in New World primates can be explained by extremely high grooming rates in the smallest monkeys, the marmosets and tamarins. These monkeys are all cooperative breeders where all family members share in taking care of infants. Humans are also considered as cooperative breeders [22]. It has been hypothesized that cooperative breeding, by requiring complex coordination among several caregivers, leads to greater communicative complexity and prosocial behavior [23,24]. An understanding of language evolution requires examining many nonhuman primate models, including cooperatively breeding monkeys where similar social and breeding systems may result in converging evolutionary processes relevant to language and cognition.

**Figure 1:**
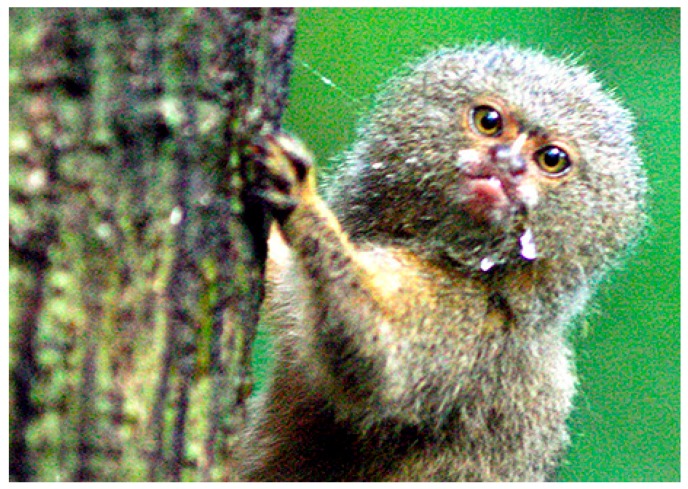
Wild pygmy marmoset feeding on exudate (Photo: Pablo Yepez).

What follows is a review (with presentation of some new data) of the cognitive components of vocal communication in cooperative breeders, using as an example, the world’s smallest simian primate, the pygmy marmoset (*Cebuella pygmaea*). Pygmy marmosets live in the Western Amazon in Ecuador, Colombia, Bolivia, Brazil, and Peru. They eat fruits and insects but a major part of their diet is excavating holes in trees with specially adapted incisors to consume exudates (see Figure 1). They live in small family groups where group members share in care of new born infants. The mean body weight of adult pygmy marmosets is 97.1 ± 22.2 g with a mean brain weight of 4.5 ± 0.6 g. [25].

I will review findings in five different areas based on the research of my colleagues and I that have implications for understanding cognitive precursors for language evolution: (1) Flexibility of calls with distance from others; (2) call convergence with new groups or mates; (3) dialects in wild populations; (4) babbling behavior by infants and adults and (5) food associated calls.

## 2. Flexibility of Call Structure with Distance

Pygmy marmosets have several contact calls that are used as animals move through their environments. In captivity, we identified four types of trill vocalizations (high pitched, sinusoidally frequency-modulated calls) that differed in frequency range, duration, and whether they were continuous or interrupted. Based on their acoustic structure we hypothesized each call type provided different cues of sound localization. High pitches, minimal frequency modulation, and continuous calls are difficult to localize, whereas calls that have multiple notes and cover a broader frequency range would be easier to localize. If this hypothesis is correct, then we would predict that calls that are difficult to localize would be best used when animals are close together, and calls with multiple cues for sound localization should be reserved for when animals are relatively far apart, assuming pygmy marmosets have some control and flexibility over which vocalizations they use.

To test this hypothesis we studied a single group of wild pygmy marmosets in Peru [26]. Whenever an animal gave one of these trill-like calls, we recorded it and immediately looked to determine the location of the nearest visible recipient. We then calculated the distance between the caller and nearest recipient. There was a close correspondence between the use of specific calls and the distance between caller and recipient with the most cryptic calls given when monkeys were usually within 5 m of each other and the more easily locatable calls given when animals were furthest apart with intermediate calls given at intermediate distances. Thus, the pygmy marmosets appeared to be using the calls strategically to maximize audibility while minimizing the likelihood of detection by predators.

In a later field study in Ecuador, Stella de la Torre replicated and extended these results to multiple groups [27]. An additional call (Type B alerting call [28]) was used for longer distance communication than we had studied before. Furthermore, de la Torre broadcast calls and pure tones through the forest where the marmosets lived and rerecorded these calls at different distances from the speaker. The call used for short range communication was highly distorted at 10 m distance and not audible at 20 m, whereas the long distance call had minimal distortion and was the only call that could be detected at 40 m. Thus, not only does the call structure change with increasing distance between caller and recipient, but the clarity and audibility of the calls changes as well.

A recent study of captive common marmosets (*Callithrix jacchus*) has shown these monkeys also change call structure as a function of distance from and visibility of their mate [29]. With greater distance and reduced visibility marmosets used different calls. Call rate, pitch, and amplitude all decreased as animals were closer to each other. Since different calls could be due to different levels of arousal, this study also measured heart rate as a proxy of arousal. Heart rate in general increased with greater distance from the mate. However, heart rate was also moderated by the timing of a mate’s vocalization as were call structures. Marmosets showed less arousal and gave the calls used in close contact if the mate responded quickly, and they showed greater arousal and the use of calls given at greater distances if the mate did not respond.

Pygmy marmosets also showed responsiveness to the calls of other group members. In a study of captive pygmy marmosets we found a clear turn taking convention with each animal calling in sequence more likely than an animal calling twice before others had called [30]. Furthermore, one sequence of turn taking among three animals (1,2,3) was significantly more common than the other possible sequence (1,3,2). These pygmy marmosets were able to identify each of the others and had developed a turn-taking rule to coordinate calling among themselves. Turn taking has also been reported in gestural communication in chimpanzees and bonobos [31].

Common marmosets and pygmy marmosets are constantly monitoring the location of others and are sensitive to the responses of other group members, and they show this both by adjusting the acoustic structure of their calls and by sequencing the timing of calls which provides a compromise between communicating with conspecifics and minimizing eaves-dropping by predators. These appear to be clear examples of both vocal flexibility as well as the strategic use of calls as influenced by both the physical and the social environment.

## 3. Call Convergence

One of the most compelling examples of vocal learning in vertebrates has been seen in song birds. Isolate-reared birds do not acquire normal song, but if played tape-recordings of song at a critical developmental stage, they go on to acquire adult song [32]. In other studies, the critical period can be extended through exposure to live tutors [33] and in still other species there appears to be learning of new songs throughout life (as in [34]). In nonhuman primates, where monkeys have been deafened or isolated from other conspecifics from birth, the results have indicated little or no effects on vocal production (squirrel monkey, *Saimiri sciureus* [35,36], rhesus macaque, *Macaca mulatta* [37]) leading to the conclusion that nonhuman primates show no evidence of vocal production learning [15,16], although they do show an ability to learn appropriate usage and responses to calls. Today it would be considered unethical to deliberately deafen or keep a monkey in social isolation, so alternative methods need to be used to evaluate the potential for vocal learning in primates. In this and the next two sections I consider three demonstrations of vocal production learning or structural flexibility in pygmy marmosets: call convergence, presence of dialects, and babbling behavior reinforced by caregiver attention.

We have documented individual variability in call structure and have shown that marmosets respond differentially to individual specific calls in playback trials [38]. We received a second group of pygmy marmosets from another facility, recorded their vocalizations, and found group differences in call structure as well. We recorded trill vocalizations in both our resident marmosets and, during quarantine (with acoustic isolation from our residents), from the new groups. After the quarantine period, we moved the new groups into the same colony room as our resident marmosets, but kept each group in separate enclosures, although they had acoustic contact with one another. Over the next six weeks we recorded the trill vocalizations of both residents and newcomers and found that individuals converged on a new trill structure that differed from the previous acoustic parameters of both the residents and the newcomers [39]. Calls changed within the first three weeks after putting all groups in the same colony room. There were no differences as a function of age with both younger and older animals showing similar changes in trill structure. It appeared that all groups of animals rapidly changed trill structure to create a new, common structure among all the groups in the colony room.

In a second study we recorded trill vocalizations from adult pygmy marmosets living in their families of origin and measured the parameters of their calls. Then we paired each animal with an opposite sexed mate from a different natal group and recorded calls for an additional six weeks and, in some pairs, again at three years after pairing [40]. Within three weeks we saw that one or both animals had changed parameters of their trill vocalization to match the call structure of their new mate, effectively going from a “his” call and a “her” call to “their” call. When we recorded from some of these pairs three years after pairing, we found that they still showed similar trills to each other, even though in some cases the structure had changed over time. Thus, in both studies, the marmosets changed the acoustic structure of their most common vocalization in response to a changed social environment. There was no change in husbandry, and the changes cannot be explained by maturation or any other obvious physical change. The most parsimonious interpretation is that the animals are learning to match the calls of new social partners or colony mates.

Similar results have been found with species assumed to be excellent vocal learners. In goldfinches (*Spinus tristus*) Paul Mundinger [41] recorded calls from males and females before and after pairing and found that each of the birds dropped some of their own calls and began using the calls of their mate. In greater spear-nosed bats (*Phyllostomus hastatus*) Janelle Boughman [42] showed that there were colony specific call structures and, when some bats from one colony were moved to another colony, they quickly acquired the calls of the new colony. These studies have been used to argue for vocal learning in birds and bats, and the finding of similar results with similar manipulations in pygmy marmosets should be presumptive evidence for vocal learning in this species. A recent study on captive chimpanzees claimed convergence in structure of food calls by immigrants after being integrated into an existing colony [43] so call convergence is found in other primates as well.

## 4. Dialects

In bird song different regional dialects are learned through interaction with the songs of residents [44] and the presence of dialects has been assumed to be an indication of learned vocal structure. There have been a few reports of differences in call structure between primate social groups (wild chimpanzees (*Pan troglodytes*) [45] and captive Barbary macaques (*Macaca sylvanus*) [46]) but the only evidence of population specific dialects has come from field research with pygmy marmosets [47]. A dialect is a common call structure among several different groups within a population that is separate from any group or individual variants in vocal structure. De la Torre recorded calls from two to three groups in each of five populations in Eastern Ecuador. The sampled populations covered a span of 150 km from east to west and 50 km from north to south. Although there were individual-specific and group-specific differences in the structure of the two most common calls, trills and J-calls, when these differences were controlled for, there was a clear distinction in call structure across populations. Each call type had an acoustic structure within a population that differed from each of the other four populations.

There are several potential explanations of this variation. One possibility is that call structure is modified by the acoustics of the local environment. De la Torre measured the noise spectra within the territory of each group and broadcast and re-recorded sound of different pulse rates to measure reverberation of sounds at different frequencies. There was considerable variation between populations in habitat acoustics, but this variation did not correlate to the call structure differences in each population. Thus, one might expect call structure to have higher pitches in environments where ambient noise was at a higher frequency range, but that did not occur. Or one might predict a larger gap between successive notes in habitats with greater reverberation, but that did not occur. Differences in habitat acoustics could not explain the differences in call structure between populations.

A second explanation is that calls differ due to genetic differences between populations. The Amazon basin with its many rivers provides many barriers to movement between populations. As a result genetic differences may develop over time, and these could explain call differences. We do not know which genes might be involved with vocal structure in primates, but, to date, using currently available primers, there is more genetic variation within than between populations. Genetic differences may still be important. A recent paper from Brazil [48] has described a new species of *Cebuella* based on genetic analyses.

A third and most likely explanation is that the dialects represent a cultural adaptation. As noted above, studies of captive marmosets have demonstrated changes in call structure when two colonies were combined and housed in the same room and the adoption of a common pair signature when new pairs are formed. The vocal structures of pygmy marmosets are flexible and change rapidly in response to changes in social conditions. Therefore, it is likely that dialects could have arisen through social learning from other group members. Supporting this notion is another study carried out on the same populations by Yepez and colleagues [49]. They observed that each population had a preferred species of tree that they used for extracting exudate. This could be due to differential abundance of these species in each population; however, a survey of the abundance of each species in each population showed that the preferred species for exudate feeding was never the most abundant in the area. This may also be a result of social transmission of feeding preferences within a population. If so, this suggests a cultural acquisition of both vocal structure and preferred exudate trees within a population.

## 5. Babbling

One of the most notable features of human language development is the vocal babbling behavior seen in infants prior to the ability to produce words. There are several characteristics of human babbling: 1. It is universal and frequent regardless of culture; 2. It is rhythmic and repetitive; 3. It begins early in life; 4. It contains a subset of the phonetic units used in adult speech; 5. The units that are produced have the structure of adult speech; 6. Babbling lacks apparent meaning since sounds do not correlate with contexts in which adults use the same sounds; 7. Babbling elicits social interaction from caregivers [50].

The most common animal model of babbling behavior has been song birds. Male birds go through a period of producing seemingly random and haphazard vocal phrases that eventually coalesce into the song pattern of sexually mature birds [51]. However, song is a sexually selected trait used mainly or exclusively by males to deter rivals and attract mates. Male and female birds have many other vocalizations but these have rarely been examined with respect to their developmental patterns. The babbling like behavior preceding song does not occur until puberty and is seen mainly in males so bird song is not a very useful model for human babbling which is seen early in life in both sexes. However, a recent study of Australian magpies (*Gymnorhina tibicen*) has documented an early onset to babbling behavior in both sexes and has found a progressive improvement in call structure in the days before fledging the nest [52]. Because magpies can also imitate human speech, the study also looked at acquisition of human like sounds and found developmental patterns similar to human babbling, but this is the rare finding of parallels in bird vocalizations.

On the first day of a visit to the field site where de la Torre studied pygmy marmosets in Eastern Ecuador, we were expecting disappointment since she had been unable to locate the marmoset groups for several months. However, to our surprise we quickly located one of her groups due to the loud and persistent vocal behavior of infant monkeys. Why do infants vocalize extensively, calling attention to themselves and their caregivers? In our captive colony, we had also observed this vocal behavior which we labeled Pygmy Marmoset Babbling due to the many similarities between babbling human infants and these vocal marmoset infants [53].

The behavior of infant marmosets closely paralleled the components of human babbling: All infants showed the behavior, and they did it beginning in the first week of life. The patterns were repetitive and included a subset of adult vocalizations. The calls were not as well formed as adult calls, but there was a progression with increased experience toward adult call structure. The calls given by adults in specific contexts appeared juxtaposed with other calls in babbling. Thus a food call, a threat vocalization, and an alarm might all appear within a few seconds similar to the lack of meaning in human babbling. Finally, babbling behavior elicited social interactions from caregivers just as in human infants [53]. Pygmy Marmoset Babbling does differ from what has been termed canonical babbling [50] which consists of strings of well-formed syllables. What is seen in pygmy marmosets is similar to pre-canonical babbling. Nonetheless, this type of vocal activity is unusual in nonhuman primates.

Does babbling have any function relative to vocal development? Infants that babbled more and used a greater variety of calls in their babbling in early infancy produced more adult-like call structures shortly after weaning [54]. Thus, greater practice at babbling seemed to promote adult-like calling sooner than those who babbled less. A recent study on common marmosets has also described babbling behavior in infants and has shown that when adults socially reinforce specific infant call types, the infants develop those adult calls sooner [55]. A similar result has been reported with human infants where parental responsiveness to babbling leads to earlier acquisition of adult phonemes [56].

Thus, babbling behavior appears to lead to better or more rapid production of adult calls, but this still does not explain completely why infants call so loudly and extensively [53]. If they could attract our attention, they could also attract predators as well. A possible hypothesis is that this extensive vocal behavior also signals to caregivers that the infant is healthy and vigorous and thus worthy of parental care and attention. Marmosets give birth to twin infants every six months or so implying a potentially high mortality rate. An infant that can solicit more care from its family may be more likely to survive as well as learning adult calls more quickly.

Although babbling behavior is common in infant animals, we have also observed babbling being used by older animals, often in conflict situations. In field studies babbling by adult animals may appear when two arrive at the same time at an exudate feeding source or when there is other competition [57]. Adult babbling is produced by the animal that has been the target of aggression, suggesting that babbling may have a submissive function in adults, much like female herring gulls use infant vocalizations when threatened by males [58].

**Figure 2:**
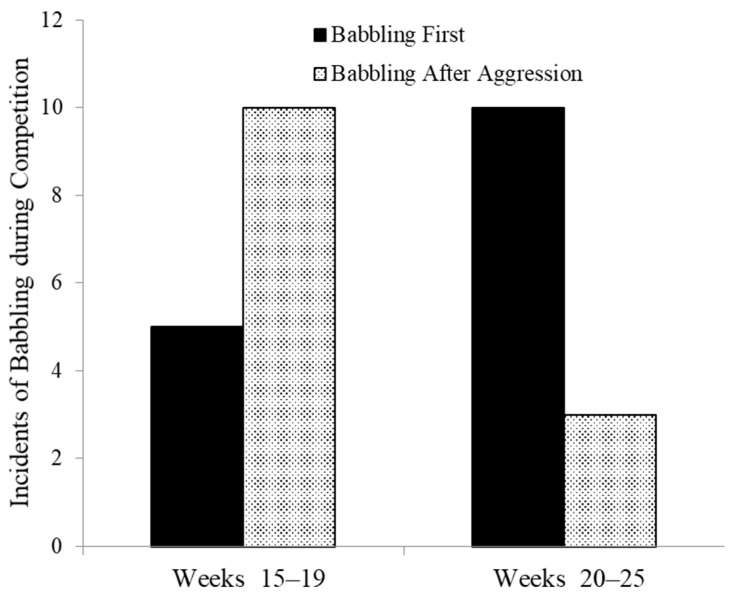
Instrumental use of babbling to deter aggression as a function of age (binomial test *p* = 0.046).

We have also observed an apparent learning of use of babbling in competitive situations. Laura Johnson studied four infants in captivity to test the development of various vocalizations. To induce food calls she presented weaned infant and juveniles with raisins, a food highly preferred by all group members. There were two interesting developmental trends. From 9 weeks of age onward through 24 weeks adults increasingly challenged infants for raisins. At first infants frequently lost, but they began to give chatters (an aggressive vocalization, see Section 6 below) when challenged. Younger infants frequently lost possession of raisins whether they called or not, but with increasing age, they showed increasing rates of chatters when challenged, and, subsequently, infants retained control of the raisins. An interesting side effect of eating raisins was that marmosets became thirsty, but with a single water bottle in the cage infants older than 14 weeks were challenged by adults when they sought access to water when an adult was nearby. From 15–19 weeks of age young marmosets were often targets of aggression at the water tube and usually produced babbling after receiving aggression. Rarely did they babble before approaching the water. However, between weeks 20 and 24 the same infants developed an instrumental use of babbling, engaging in babbling upon approach to an adult at the water bottle rather than waiting until they had received aggression. In these older infants babbling first occurred significantly more often that babbling afterwards (binomial test, *p* = 0.046, Figure 2).

In summary, with the exception of the Australian magpie animal model studies of babbling which have focused on birds where the babbling like behavior occurs mainly in pubescent male birds in a mating context. This is equivalent to using the courtship sounds of adolescent boys as a model for human language development. The pygmy marmoset presents a more realistic model for studying babbling behavior, occurring early and universally in both sexes, containing a subset of the adult vocal repertoire, that are not fully formed. Babbling is repetitive, and the call types are unrelated to context. Furthermore, as in human infants, babbling elicits social interactions with caretakers and this may have both a protective function and may reinforce vocal development, as seen in experimental work with common marmosets. Infants that babble more often and with greater variety reach adult call structure sooner than those who do not. Babbling is also used instrumentally to signal subordinate status or to avoid aggressive interactions, and this seems to be a learned behavior.

Much has been written about the lack of vocal learning in nonhuman primates [15,16], but the definition of learning has been quite strict: the acquisition of calls that are not part of the normal vocal repertoire. This would be difficult to demonstrate even in most song birds where vocal learning has been studied primarily. Some birds, such as parrots, mynahs, and starlings do regularly imitate calls outside of their vocal repertoire. However, the authors who argue for the absence of vocal learning in primates readily accept the results on bats [42] showing vocal convergence similar to what has been seen in pygmy marmosets. If vocal learning includes modification of call structure to match social companions, the presence of population specific dialects that cannot be easily explained by environmental or genetic factors, and the systematic shaping of vocal structure through parental reinforcement, then pygmy marmosets need to be considered as vocal production learners.

## 6. Food Associated Calls

Food-associated calls have been described in many species ranging from chickens [59] to chimpanzees [60] (see also paper by Rogers, Stewart, and Kaplan on common marmosets in this special issue [61]). Food-associated calls have been cited as a form of referential signals (that is calls that refer to specific objects beyond the caller’s own emotional state). Similar claims have been made for predator-specific alarm calls. However, for both food calls and alarm calls there are also potential emotional components: hunger or preference in the case of food-associated calls, and fear in the case of predator alarm calls. For example, chickens give more calls to high preference foods than to low preference foods [59]. Studies of cotton-top (*Saguinus oedipus*) and golden lion tamarins (*Leontopithecus rosalia*) have also found that food-associated calls are correlated to food preferences [62,63]. A study of Geoffroy’s marmosets (*Callithrix geoffroyi*) [64] found that playbacks of food-related calls led to increased foraging and feeding behavior by recipients compared with control playbacks, indicating that the calls meet the criteria for functional reference [65]. Thus, the calls are communicating about the presence of food even though there is a clear motivational component to the calls as well.

No published work has described or evaluated food-associated calls in pygmy marmosets. Two of my former students, Ann Fjellstad and Rebecca Addington carried out studies on food-associated calls in pygmy marmosets that are reported here. To determine that a call is specifically related to food, it is important to know that a call is not used in other contexts as well. For example, a study of toque macaques (*Macaca sinica*, [66]) found that most of what were identified as food calls were given to food but that 3% of the calls were given to positive changes in weather—to the first rain clouds at the end of the dry season and the first cloudless day at the start of the dry season—, suggesting an alternative hypothesis of elation calls rather than food calls. Marler and colleagues [59] found that chickens responded with food calls to empty peanut shells; although at a lower rate than to shelled peanuts. Thus, putative food calls may have other functions or may also be responses to inedible objects associated with food.

A description of the vocal repertoire of pygmy marmosets [28] reported three calls associated with feeding: Type B alerting call, chatters, and squeals. Furthermore, squeals occurred both as single calls and as a series of calls. Thus the first step was to determine if each or any of these calls was found only in a food-related context.

Ann Fjelstad tested seven adult pairs of marmosets. One animal died during testing so the data are based on 13 animals. Each monkey was tested with five different types of foods presented two at a time to evaluate preferences, using an apparatus that allowed a monkey to make a single choice among two alternatives (described in [62]). Ann tested marmosets with peaches, hamburger, live mealworms, and the regular marmoset diet as well as marshmallows, a novel food. Each pair of foods was presented twice to each individual (one on each side of the apparatus) and preference rankings were obtained for all foods. After preference testing was completed, the five foods, along with two nonfood manipulable control objects (paper clip and knotted string) were presented once to each pair on separate days. Vocalizations and behavior were recorded for five minutes before and after food or object presentation. Calls were identified using real time sound spectrum analysis.

The Type B alerting calls occurred with equal frequency during both food trials and in control periods and, therefore, were rejected as food-associated calls. In wild pygmy marmosets these calls appear to function as long distance cohesion calls. Chatters were common during feeding but were given when one animal chased another or when a monkey approached an animal holding food leading to the withdrawal of the approaching monkey. Thus, these calls seem to function as threats rather than food signals.

**Figure 3:**
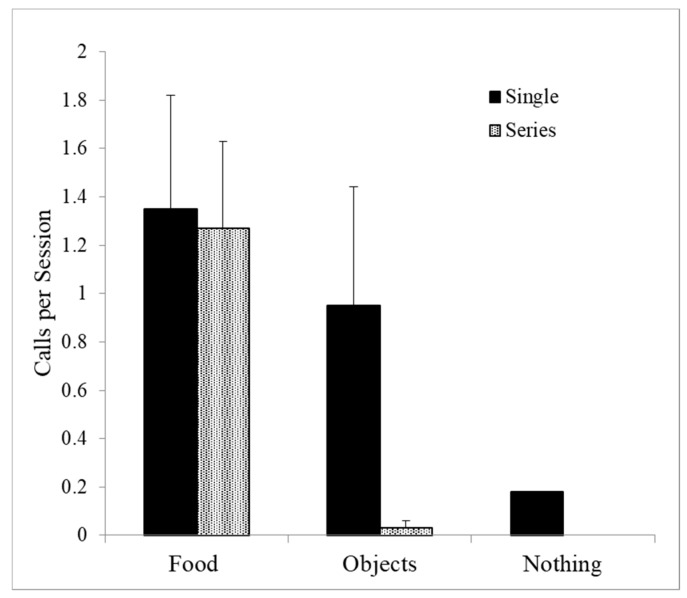
Squeal series were produced almost exclusively in feeding contexts (*p* < 0.001), whereas single squeals were given as well to nonfood items and in the absence of any objects.

This leaves only squeals as a potential food-associated call. Single squeals were given to food but also equally to nonfood manipulable objects (Wilcoxon Test, *T* = 22, *NS*). Single squeals were also given occasionally in the absence of any object, and so single squeals are also not good candidates for food-associated calls. However, series of squeals were given on food trials, but rarely on nonfood trials (Wilcoxon Test *T* = 0, *p* < 0.001), suggesting that these series are best described as food calls (see Figure 3).

**Figure 4:**
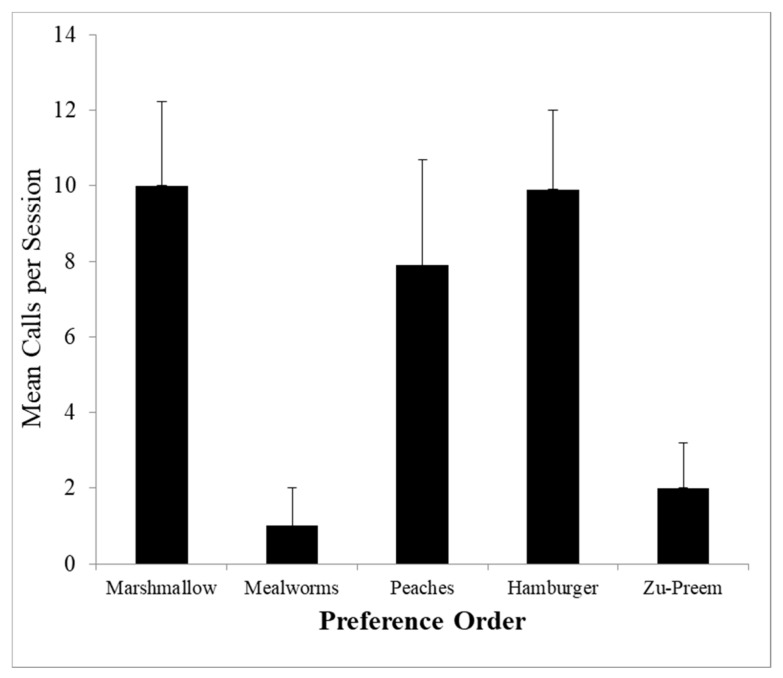
Mean rate of squeal series calling (+S.E.M.) in response to food is unrelated to food preference (*R_S_* = 0.30). Foods are ordered from most preferred on left to least preferred on right.

The marmosets exhibited clear food preferences favoring novel marshmallows first, then meal worms followed by peaches, hamburger, and least of all the marmoset chow. However, in contrast to studies relating food preferences to rate of calling in tamarins, the pygmy marmosets showed no clear relationship of number of squeals produced and food preferences (*R_S_* = 0.300, *p* = 0.624, Figure 4). This was in part due to an inhibition of calling when live mealworms were presented. A similar finding was reported with wild white-faced capuchin monkeys (*Cebus capucinus*) in Costa Rica [67]. Capuchin monkeys gave more than 4 times as many calls while foraging and feeding on fruit than they did while foraging on insects. Most other studies of food calls have not compared live prey versus other foods so we do not know if this would result would hold with other species, but it appears that pygmy marmosets and capuchin monkeys are suppressing food calling in the presence of live prey. Since live prey may sense the approach of a predator and escape, food call suppression is an adaptive foraging technique. These findings show that food associated calls are not simple reflexive calls given in response to all edible food, but rather, pygmy marmosets (and capuchin monkeys) use these calls strategically. However, field observations [68] have found squeals are given to live prey rather than to exudate, suggesting potential differences between captive and field populations.

Some have questioned why any species should give food calls that alert other group members to food. Should not an animal behave selfishly and protect its own interests? A study of rhesus macaques (*Macaca mulatta*, [69]) found that monkeys that failed to call upon finding food were punished by others. In cooperative breeding species, however, we have not seen any indication of punishment, but since all family members work together to raise infants, sharing food appears to be adaptive, and tamarins communicate honestly about their most preferred foods.

**Figure 5:**
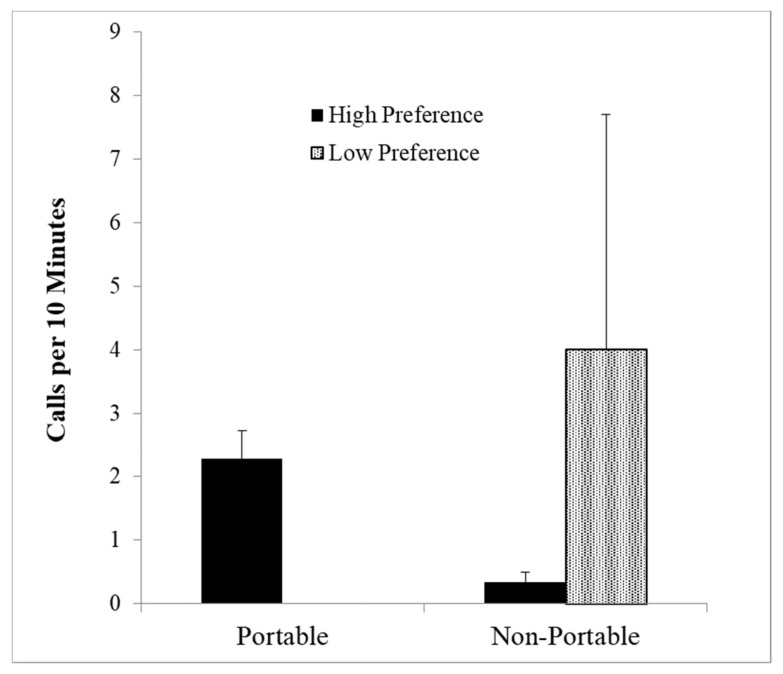
More squeal series given to high preference portable foods but to low preference non portable food (*F* = 36.99, *p* < 0.001).

Portable or sharable resources may also affect food calling. In studies of chimpanzees [70], animals would not call to small amounts of food (prunes) or to a large nonsharable food source (whole watermelon). However, if the watermelon was cut into several pieces, chimpanzees would give food calls. This suggests that sharable foods may lead to more calls than nonsharable foods. To test this in pygmy marmosets Rebecca Addington [71] presented 19 pygmy marmosets (8 in mated pairs; 11 unmated animals) with clumped versus distributed food, where food was either portable (pieces of hamburger (high preference) and jelled marmoset jelly (low preference)) or nonportable (liquefied hamburger and liquid marmoset jelly). She found an increase in aggression when foods were clumped, and a linear relationship between chatter calls and likelihood of aggression (*r*(17) = 0.91, *p* = 0.002). This is further validation that chatter calls are related to aggression, rather than food. Squeal series showed a significant interaction between portability and food preference (*F* (1, 6) = 36.99, *p* < 0.001) with squeals given only to the high preference food (hamburger) in the portable condition, and significantly more to the low preference food (marmoset jelly) in the nonportable condition (Figure 5).

**Figure 6:**
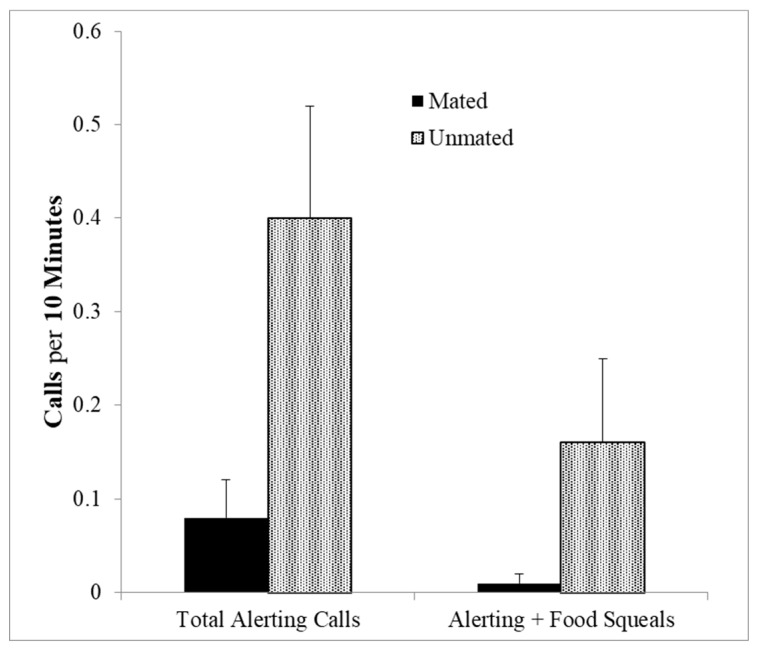
Both alerting calls alone and alerting calls combined with food calls given by unmated compared with mated marmosets (*p’s* < 0.04).

Addington also observed several call sequences involving the long distance alerting calls. These calls occurred alone, or in sequences preceded by J-calls or squeals. The mean interval between adult calls is greater than 4 s whereas in the sequences, the mean time interval was short (*M* ± *SEM* = 293.4 ± 27 ms) suggesting that these really are sequential calls. Combination calls involving squeal series and alerting calls were given significantly more often by unmated than by mated animals (*U*-test (8,11), *z* = 2.05, *p* = 0.04, Figure 6). This was also the case with total number of alerting calls whether in sequence or not (*U*-test (8,11), *z* = 2.10, *p* = 0.035). This is a curious finding, but since the alerting calls are used for long distance communication in the wild, perhaps unmated individuals are soliciting potential mates and indicating a willingness to share food, by sequencing squeal series with the alerting calls. This would indicate a strategic use of food calls and long distance calls to recruit potential mates.

In summary, like many other species, pygmy marmosets have several calls that are associated with food. However, it has been necessary to distinguish among four calls commonly associated with food to find the one type of call (squeal series) that is specific to food-related contexts. Although food calling has been related to food preference in some other species, this was not the case with pygmy marmosets, due in part to the failure to call to live prey. Live prey has rarely been used in previous studies of captive animals, but the ability to suppress calls to live prey suggests awareness that different foods may require different strategies. Since animals give more calls to foods that can be shared, it is also possible that live mealworms are viewed as non-sharable. However, since there were always two pieces of each food per group member at the start of a trial, there would be more mealworms than any one individual could monopolize, suggesting that call suppression is a function of food type rather than of food abundance. Food calls were more frequent with high preference portable foods and low preference nonportable foods. This is a curious result but it may be that the calling animal is able to control a portable high preference food and is in little danger of losing it, whereas an animal that calls with a nonportable food risks having to share it with others and so vocalizes only to low preference foods. This would imply a strategic use of food calls under circumstances that would benefit the caller. Finally, the combination of food calls with long distance calls only in unmated animals suggests that food calls may also serve as a mechanism to attract mates. In each of these cases there has been a cognitive component involving strategic choices and decisions rather than simple reflexive vocalization in all situations where food is present. It is important to note that we were unable to do playback studies and, therefore, do not know if marmosets would respond with food related responses to the calls. Thus, we cannot claim that these calls are functionally referent [72]. However studies of Geoffroy’s marmosets [64] and common marmosets [61] have documented functional reference in similar calls, suggesting pygmy marmoset calls would be similar.

## 7. Discussion

Pygmy marmosets are the world’s smallest simian primates with correspondingly small brains and yet they show several aspects of cognitive complexity in their communication. This paper has reviewed research showing that pygmy marmosets systematically change the structure of their contact calls as a function of their distance from other group members. Calls that are difficult to localize and that do not travel well through the habitat are used only when animals are close to one another, whereas calls that transmit better through the habitat and that provide more cues of localizing the caller are used only when animals are relatively distant from each other. Such systematic variation in call structure with distance serves to minimize cues that predators might use to locate the marmosets. This also implies some awareness by the caller of where it is located relative to other group members, and the ability to alter call structure in a flexible manner. In addition, marmosets show distinct turn-taking patterns (antiphonal calling), which implies recognition of individuals and adoption of a turn taking rule for social communication.

Pygmy marmosets alter the structure of their vocalizations when exposed to novel social environments. When two colonies of marmosets each with their own structure of trill vocalizations were housed in the same colony room, the individuals of each colony changed some acoustic parameters of their calls within a few weeks to develop a new version of trills. Similarly, when a male from one family was paired with a female from another family, the newly formed pair converged on a single form of trill, and this convergence lasted for at least three years. The ability to match call variables of a new group is seen in human language (called optimal convergence) as well [73] and is thought to help a newcomer become better integrated into a group or population. The sharing of a common vocal structure may serve as a way to strengthen pair bonds within a mated pair and to create group solidarity within larger social units. A logical result of call convergence within groups is seen in the presence of population-specific dialects seen in wild pygmy marmosets in different locations in the Amazon. The acoustic differences cannot be explained by differences in habitat acoustics, since the call structure in each population did not directly match predictions based on habitat acoustics. Although genetic differences cannot yet be ruled out, given the evidence of vocal flexibility seen in captive studies, it seems likely that the dialects observed are the result of social learning and cultural transmission.

These results indicate a hierarchy of identification features within the calls of pygmy marmosets. There is evidence of individual recognition based on calls as seen in playback studies and in turn taking [30,38]. There are also group specific differences most likely developed as newly formed pairs adjust their calls to one another [40], but several groups of animals within the same population develop dialects that transcend group differences as seen in captivity [39] and the wild [47].

Part of the argument against vocal learning in nonhuman primates also has been based on early studies where isolate-reared or deafened monkeys have shown few deficits in communication. However, such studies would be considered unethical today and so other approaches are needed. Pygmy marmosets display a babbling like behavior that has several parallels to human infant babbling [53]. Key among the parallels is that babbling elicits social interaction with caregivers. Infants that babble more in the first month of life show more rapid development of adult vocalizations [54]. Recent work on common marmosets has shown that this social interaction with caregivers is an important mechanism for acquiring adult vocalizations and eliminating inappropriate calls [55]. Because of the widespread belief that nonhuman primates do not show vocal learning, they are often ruled out as models in which to study parallels to human language. However, research on vocal development in pygmy marmosets and common marmosets along with studies on vowel production in macaques and baboons and recent research on great apes suggest that nonhuman primates can serve as models for understanding aspects of language development and evolution [13,17].

Food calls have often been linked with predator-specific alarm calls as examples of referential signals. However, caution is needed in treating food calls as equivalent to predator-specific alarms [72]. Many of the food calls described in other species should really be labeled food-associated calls, since they may not be exclusively related to food, but rather to preference or arousal or may occur in other, nonfeeding circumstances. The data from pygmy marmosets showed that of the four calls initially recorded in feeding contexts [28] only one was specific to food, the squeal series. The other three calls were associated with different or much broader contexts. Furthermore, food calling cannot be related to arousal or preferences since there was no relationship between preference ranks and rate of food calling. Thus, the squeal series appear to be a true referential call for food. However, the data also showed that food calls are not simple reflexive responses to the presence of food. Calls were suppressed in the presence of live prey, a finding also seen in wild capuchin monkeys [67]. Some possible functions of food calls are suggested by the interaction of call rates to portability and preference. Marmosets called more to high quality portable food as well as to low quality portable food. Aggressive calls were also found frequently in feeding contexts, especially with clumped distributions [71]. Taken together, these results suggest that for pygmy marmosets, food calls indicate possession of food and may deter competition. However, since these are cooperative breeders where all group members work together to rear infants, calling also indicates the availability of food to others. This may explain the increase in calling to low preference food that is nonportable. The combination of a long distance call with a food call used only by unmated suggests that food calls may also function to draw attention to an important resource to a potential mate.

Although the pygmy marmoset has a very small brain, it is capable of cognitive complexity in its vocal behavior. They selectively produce contact calls with different structures to stay in contact with other group members. To show strategic flexibility in the use of contact calls pygmy marmosets must be able to monitor their own location and that of other group members. This is also demonstrated by a turn taking convention.

Several lines of research suggest the potential for vocal learning beginning with babbling behavior that is reinforced through social responses by caregivers, but also including call structure convergence. The presence of dialects in different populations of wild pygmy marmosets also suggests vocal learning, especially since differences in habitat acoustics cannot explain the population differences. Recent work on captive common marmosets confirms the idea of vocal learning in these species. Finally, pygmy marmosets show great flexibility in the use of food calls. Squeal series appear to be specific for food, but marmosets suppress these calls when feeding on live prey and show an interaction in call rates between portability and preference.

Each of these findings illustrates an aspect of cognitive complexity in vocal communication. Pygmy marmosets are not unique among primates in the cognitive complexity of communication, but these findings among cooperatively breeding monkeys also support the notion that cooperative breeding may lead to converging evolutionary processes in social behavior and communication that are as valuable for understanding human social behavior and the evolution of language as studies of great apes.
